# Multifunctional property exploration: Bi_4_O_5_I_2_ with high visible light photocatalytic performance and a large nonlinear optical effect†

**DOI:** 10.1039/c8ra08984a

**Published:** 2019-02-05

**Authors:** Ruonan Yin, Yang Li, Kangdi Zhong, Hang Yao, Yamin Zhang, Kangrong Lai

**Affiliations:** Department of Physics, Changji University 77# North Beijing Road Changji 831100 China laikr0212@163.com +86-889-9049560 +86-994-2336271

## Abstract

Exploration of the versatility of materials is very important for increasing the utilization of materials. Herein, we successfully prepared Bi_4_O_5_I_2_ powders *via* a facile solvothermal method. The Bi_4_O_5_I_2_ photocatalyst exhibited significantly higher photocatalytic activity as compared to the common BiOI photocatalyst in the degradation of methyl orange, methylene blue and rhodamine B under visible light irradiation. Especially, for the degradation of methyl orange, the photocatalytic activity of Bi_4_O_5_I_2_ is about 10 times that of BiOI. Moreover, Bi_4_O_5_I_2_ exhibits an extremely high second harmonic generation response of about 20 × KDP (the benchmark) estimated by the unbiased *ab initio* calculations. The coexisting multifunction of Bi_4_O_5_I_2_ is mainly because of the increased dipole moment due to the stereochemical activity of lone pairs that promotes separation and transfer of photogenerated carriers, then enhances the photocatalytic activity and results in a high second harmonic generation response. This indicates that Bi_4_O_5_I_2_ may have good potential applications in photocatalytic and nonlinear optical fields.

## Abstract 2

Bi_4_O_5_I_2_ exhibits an extremely high second harmonic generation response and enhanced photocatalytic activity. The multifunction of Bi_4_O_5_I_2_ is mainly resulting from the dipole moment of the stereochemical activity of Bi 6s lone pairs.

## Graphical abstract

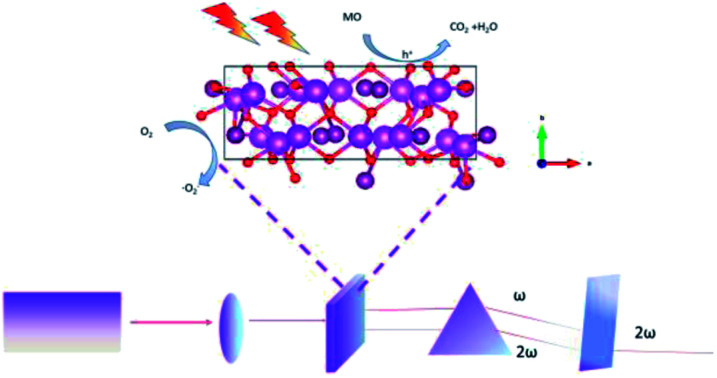

## 1. Introduction

Green, efficient, harmonious and civilized society is the development trend of future life. Ever since Fujishima and Honda have reported the photocatalytic water splitting on the TiO_2_ surface,^1^ TiO_2_ has been extensively studied for the degradation of pollutants or splitting of water.^2^ However, the wide bandgap (3.0–3.2 eV) of TiO_2_ limits its application range. Therefore, it is essential to find visible light-driven photocatalysts. Methylene blue (MB), methyl orange (MO) and rhodamine B (RhB) are the main components in common water pollutants. Scientists have invented many methods for the degradation of organic pollutants.^3–8^ Bismuth-containing materials are attracting significant attention of researchers due to their unique electronic, magnetic, optical, and catalytic properties;^9–13^ however, the existence of high photogenerated carrier recombination rate restricts the development of Bi-based photocatalysts.

To enhance the photocatalytic activity, scientists have carried out a lot of research and exploration.^14–21^ To the best of our knowledge, in 2002, Bi_4_O_5_I_2_ was successfully synthesized by changing the stoichiometry of bismuth oxyhalide.^22^ After this, non-stoichiometric ratio halogenated bismuth compounds, for example, Bi_4_O_5_I_2_, Bi_4_O_5_Br_2_, and Bi_5_O_7_Br, have been studied constantly.^23,24^ Ye *et al.* have committed to the research and development of non-stoichiometric Bi-based photocatalysts. They made a series of innovative achievements such as photocatalytic water splitting for hydrogen (H_2_) evolution,^25^ selective photoreduction of CO_2_ into CO,^26^ molecular oxygen activation and RhB degradation.^27,28^ Xiao *et al.*^29,30^ have successfully synthesized Bi_4_O_5_I_2_ nanoflakes, which had better photocatalytic activities in the degradation of 4-*tert*-butylphenol, and predicted that they could be applied to other fields as well.

Non-stoichiometric ratio halogenated bismuth compounds have become the most promising second-order nonlinear optical (NLO) and photovoltaic (PV) candidate materials owing to their special structural features, rich chemical compositions, and suitable optical band gaps. In fact, most of these noncentrosymmetric structures could be applied in more than one of the aforementioned fields. It is known that nonlinear optical (NLO) materials have played an important role in laser science and technology field and are rapidly developing.^31–34^ The NLO crystals are laser frequency conversion materials, and their SHG coefficients are the focus of attention, which may reflect the ability of the NLO crystal to generate harmonic laser emission.^35,36^ Among them, fluoroborate is responsive to ultraviolet light and is widely used in military, medical, aviation and other fields.^37^ It is known that lower SHG coefficient limits their applications. Therefore, the search for new NLO materials with SHG response larger than 10 × KDP has attracted significant attention.

In general, noncentrosymmetric crystal structure materials may possess very good NLO properties.^28^ In 2014, Fan *et al.*^38–40^ found a series of materials, such as K_3_B_6_O_10_Br, Na_3_VO_2_B_6_O_11_, and M_2_B_5_O_9_Cl (M = Ca, Sr, Ba, Pb), with noncentrosymmetric structures. Due to intrinsic large polarization effects, they not only have good nonlinear optical properties, but have also been found to exhibit significantly enhanced photocatalytic activities.

To the best of our knowledge, the noncentrosymmetric polarized Bi_4_O_5_I_2_ belongs to the space group *P*2_1_.^22^ Then, does it have the multifunctional property? To research the potential properties of Bi_4_O_5_I_2_, we carried out photodegradation experiments and theoretical calculations of the optical properties. The results showed that Bi_4_O_5_I_2_ had high visible light photocatalytic performance. Moreover, the theoretical calculation showed that Bi_4_O_5_I_2_ exhibited large second harmonic generation (SHG) response. At present, the versatility of materials is a hot research topic that scientists have been exploring. Based on the abovementioned findings, herein, we successfully prepared BiOI and Bi_4_O_5_I_2_ materials, and some characterizations and calculations were performed to verify the versatility of these materials.

## 2. Experimental

### 2.1. Materials and methods

Ethylene glycol (EG), bismuth nitrate pentahydrate (Bi(NO_3_)_3_·5H_2_O), and potassium iodide (KI) were purchased commercially and used without further purification. Moreover, deionized water was used throughout this study.

Bi_4_O_5_I_2_ powders were synthesized *via* a facile solvothermal method. A mixture of 2 mmol Bi(NO_3_)_3_·5H_2_O, 4 mmol KI and 35 mL EG was prepared and transferred to a 45 mL Teflon-lined stainless steel autoclave, and the pH of the suspension was adjusted to 10 using 2 mol L^−1^ NaOH. Subsequently, it was maintained at 150 °C for 12 h followed by naturally cooling to room temperature. The precipitate was centrifugally washed with distilled water and absolute ethanol for several times and then dried at 80 °C for 5 h under an air atmosphere. BiOI was obtained through the abovementioned procedure without the addition of NaOH.

### 2.2. Characterization

Phase identification was performed using the Bruker D8 ADVANCE X-ray diffractometer (XRD) equipped with a diffracted-beam monochromator set for Cu and Kα (*λ* = 0.154059 nm) as the radiation sources. The acceleration voltage and acceleration current are 40 kV and 40 mA, respectively. The results were obtained by scanning the sample through a range of 2 theta values (10–85°). UV-visible diffuse reflectance spectra (DRS) were obtained *via* the Shimadzu UV-3600 UV-vis spectrophotometer using BaSO_4_ as a reference. The morphologies of the samples were observed *via* a scanning electron microscope (SEM) using a Zeiss SUPRA 55VP apparatus, and energy dispersive X-ray (EDX) spectroscopic analysis was conducted using EDX-8000. The photocurrents (PC) of UV-vis light on and off studies were measured *via* the CHI660E electrochemical system (Shanghai, China) using a standard three-electrode cell containing a working electrode (1.0 cm × 1.0 cm), a platinum plate as the counter electrode, and a standard calomel electrode (SCE) as the reference electrode. Electrochemical impedance spectroscopy (EIS) measurements were carried out using an electrochemical workstation (Zahner Im6ex) with the working electrode in the frequency range from 100 kHz to 0.01 Hz at open circuit potential in a 0.1 M KCl solution.

### 2.3. Photocatalytic reaction

The photocatalytic activities of BiOI and Bi_4_O_5_I_2_ samples were tested using a 300 W Xe lamp as the light source and by the degradation of MO, MB, RhB and norfloxacin (NOR) under the visible-light irradiation. Optical filters were used to eliminate the UV light (*λ* ≤ 420 nm). The power density at the position of the reactor is about 7.4 mW cm^−2^. In each of the experiment, 50 mg catalyst powder was dissolved in an aqueous solution of MO (100 mL, 10 mg L^−1^) in a beaker. Prior to irradiation, the suspensions were magnetically stirred in the dark for 30 min to achieve an adsorption–desorption equilibrium. Reduction in the concentrations of dyes was analyzed by the Cary 500 UV-vis spectrophotometer at the wavelength of maximal absorption (464 nm, 664 nm, and 554 nm). The samples of the reaction solution were taken out and then centrifuged and filtered at regular intervals. Finally, the filtrates obtained were analyzed.

### 2.4. Numerical calculation details and methods

The electronic structures and NLO properties of BiOI and Bi_4_O_5_I_2_ were simulated by the CASTEP package.^41^ The experimental crystal data were used in density functional theory (DFT) calculation *via* a plane wave pseudo-potential method. During the calculation, geometry optimization was performed using the BFGS minimization technique. The local density approximation (LDA) with the Ceperley–Alder–Perdew–Zunger functional and the ultrasoft pseudopotentials (USPs) were chosen as the exchange correlation functional and pseudopotential, respectively.^42^ In the electronic structure and optical property calculations, the ultra-fine pseudopotentials were set up for BiOI and Bi_4_O_5_I_2_. Bi 5d^10^6s^2^6p^3^, I 5s^2^5p^5^, and O 2s^2^2p^4^ were chosen as the valence electrons. The *k*-point separation of each material was set to 0.04 Å^−1^ in the Brillouin zone.

## 3. Results and discussion

### 3.1. XRD analysis

**Fig. 1:**
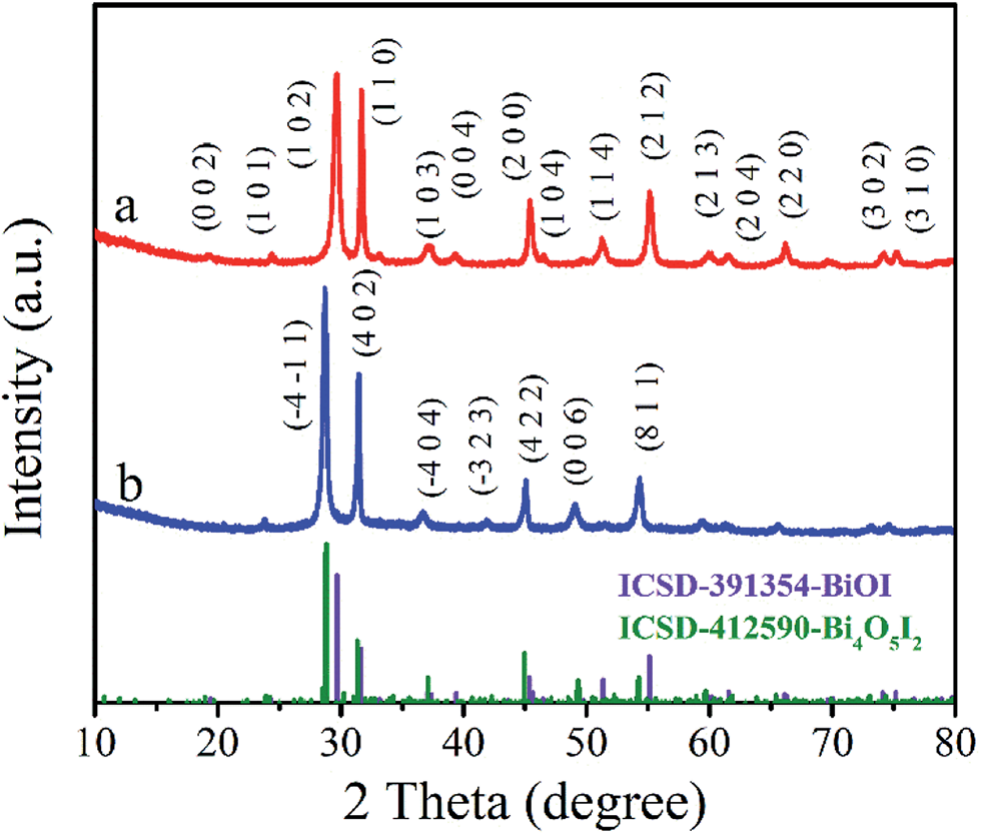
XRD patterns of the BiOI (a) and Bi_4_O_5_I_2_ (b) samples.

BiOI and Bi_4_O_5_I_2_ belong to the tetragonal space group *P*4/*nmm* with cell dimensions *a* = 3.995(2) Å, *b* = 3.995(2) Å, and *c* = 9.151(5) Å and noncentrosymmetric monoclinic space group *P*2_1_ with cell dimensions *a* = 14.944(1) Å, *b* = 5.6983(3) Å, and *c* = 11.263(1) Å, respectively. Powder XRD was used to characterize the crystal structure of the products. Fig. 1 shows the XRD patterns of the BiOI and Bi_4_O_5_I_2_ samples. As shown in Fig. 1a, all the observed peaks of the pattern can be indexed to the tetragonal phase of BiOI (ICSD 391354), and the observed major diffraction peaks at 29.65°, 31.66°, 45.38°, 51.35° and 55.15° belong to (102), (110), (200), (114) and (212) planes of the tetragonal phase BiOI, respectively. Fig. 1b clearly shows that the diffraction data match well with the phase of Bi_4_O_5_I_2_ (ICSD 412590). Moreover, it can be clearly seen that the characteristic peaks located at 28.8, 31.5, 45.1, 49.3, and 54.4 can be indexed to (−4−11), (402), (422), (006), and (811) planes of Bi_4_O_5_I_2_, respectively (Fig. 1b). These findings are consistent with the previously reported data. Therefore, it can be initially determined that BiOI and Bi_4_O_5_I_2_ samples have been successfully synthesized.

### 3.2. SEM and EDS analyses

**Fig. 2:**
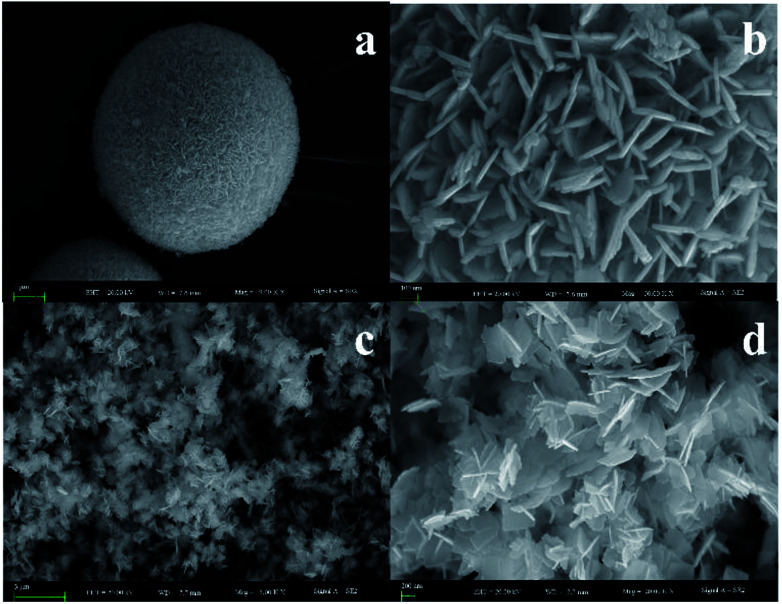
SEM images of different magnifications of BiOI (a and b) and Bi_4_O_5_I_2_ (c and d) samples.

To identify the morphological information and atomic compositions of samples, BiOI and Bi_4_O_5_I_2_ samples were investigated by SEM and EDS. The results are shown in Fig. 2. As clearly observed from Fig. 2a–d, the architecture and morphology of Bi_4_O_5_I_2_ nanosheets are distinctly different from those of BiOI. The flaked structure of the Bi_4_O_5_I_2_ nanosheet has been successfully prepared. The EDS results are shown in Fig. S1a and b,† and the Bi : I ratios of Bi_4_O_5_I_2_ and BiOI are determined to be 2.2 : 1 and 1.08 : 1, respectively. The EDS results further demonstrate the conclusion drawn based on the XRD results.

### 3.3. UV-vis diffuse reflectance spectral analysis

**Fig. 3:**
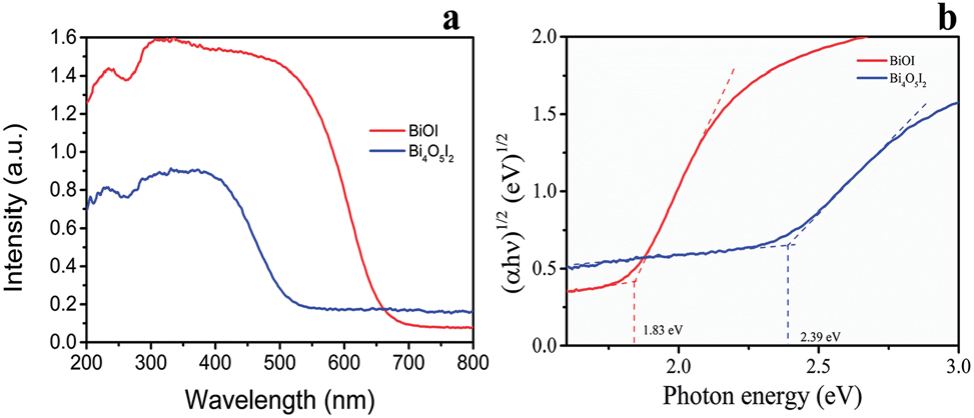
(a) UV-vis diffuse reflectance spectra of Bi_4_O_5_I_2_ and BiOI. (b) Plots of (*αhv*)^1/2^ of Bi_4_O_5_I_2_ and BiOI *versus* photon energy (*hv*).

As shown in Fig. 3a, the absorption edges of BiOI and Bi_4_O_5_I_2_ samples are about 663 nm and 536 nm, respectively. The band gap energies of BiOI and Bi_4_O_5_I_2_ may be determined by the following equation:^43–47^
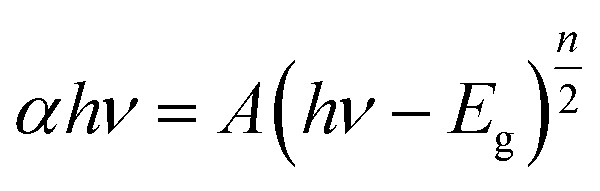
where *α*, *h*, *hν*, *A*, and *E*_g_ are the absorption coefficient, Planck's constant, photon energy, a constant value, and bandgap energy, respectively. In addition, *n* is determined by the transition type of a semiconductor (*n* = 1 for direct transition, whereas *n* = 4 for indirect transition). According to the previously reported studies,^48^ the *n* value of BiOI and Bi_4_O_5_I_2_ is 4, which is consistent with the band structure results. According to the formula, the band gap energies for BiOI and Bi_4_O_5_I_2_ samples are found to be 1.83 and 2.39 eV, respectively.

### 3.4. Photocatalytic degradation analysis

As shown in Fig. S2a†, an adsorption–desorption equilibrium has been achieved in dark for 30 min. To better understand the photocatalytic effect of the samples, MO solutions at different initial concentrations were used for comparison. It is observed from Fig. S2b† that lower concentrations of MO degrade more efficiently. As the initial concentration of MO increases, additional organic molecules are able to adsorb onto the photocatalysts surface, thereby reducing the photogeneration of the reactive oxygen species. In addition, it can be clearly seen that Bi_4_O_5_I_2_ displays relatively better degradation efficiency than TiO_2_ (P25) (Fig. S2a†).

Herein, MO, RhB and MB dyes were selected as model pollutants to evaluate the photocatalytic activities of BOI and Bi_4_O_5_I_2_ (Fig. S2c†). It is clearly seen that Bi_4_O_5_I_2_ displays relatively better degradation efficiency than BiOI. Moreover, as can be seen, under the light irradiation for 100 min, about 29%, 50%, and 90% of RhB, MO and MB were degraded over the BOI samples, respectively. However, under the light irradiation for 60 min, 94% of MO and MB were degraded over the Bi_4_O_5_I_2_ samples. Note that under the light irradiation for 40 min, 99% of RhB was degraded over the Bi_4_O_5_I_2_ samples. In Table ESI-1,† the results show that the photocatalytic activity of the Bi_4_O_5_I_2_ photocatalyst is about 10 times that of BiOI for the degradation of MO. In addition, to minimize the effect of dye sensitization, degradation of colorless solution has been performed (Fig. S2d†). As can be seen, under light irradiation for 100 min, about 43% of NORs were degraded over the Bi_4_O_5_I_2_ samples.

### 3.5. Free-radical trapping experiment analysis

Trapping experiments may confirm the main active species during the photodegradation process.^4,30^ Ar, IPA, and KI purging were employed to quench the active species such as ·O^2−^, ·OH and h^+^, respectively. At first, trapping agents were dissolved in a Bi_4_O_5_I_2_ solution before the reaction. The free-radical trapping experiment results are shown in Fig. S3a.† It can be seen that the photoinduced h^+^ and ·O^2−^ radicals are the primary reactive species. To evaluate the repeatability of the Bi_4_O_5_I_2_ sample, recycling experiments for the photocatalytic degradation of MO over the Bi_4_O_5_I_2_ sample were performed (Fig. S3b†). Note that even after 4 cycles, the photocatalytic performance of Bi_4_O_5_I_2_ was retained. These results imply the high stability of the Bi_4_O_5_I_2_ sample, which is significant for its practical application.

### 3.6. Band structure analysis

**Fig. 4:**
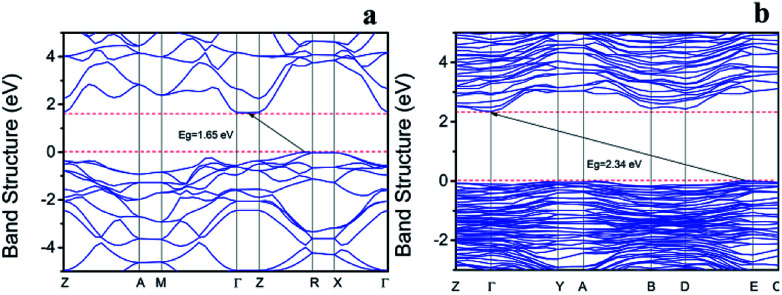
Band structures of BiOI (a) and Bi_4_O_5_I_2_ (b).

The electronic structures of BiOI and Bi_4_O_5_I_2_ were calculated. As shown in Fig. 4a, the highest valence band (VB) is located between the R and Z points and the lowest conduction band (CB) is located between Γ and Z points for BiOI. As shown in Fig. 4b, the highest valence band (VB) is located between D and E points and the lowest conduction band (CB) is located at Γ point for Bi_4_O_5_I_2_. Therefore, the calculated indirect band gaps of BiOI and Bi_4_O_5_I_2_ are about 1.65 and 2.34 eV, which are close to the experimental values of 1.83 and 2.39 eV, respectively.

### 3.7. Dipole moment analysis

To identify what promotes the effective separation of photogenerated electron–hole pairs of Bi_4_O_5_I_2_, the dipole moment calculation was carried out.^39^ The calculations of the Bi–O and Bi–I bond valences and dipole moments of the BiO_*m*_I_*n*_ (*m* = 4, *n* = 4; *m* = 5, *n* = 3; *m* = 6, *n* = 1; *m* = 3, *n* = 4; *m* = 3, *n* = 5) polyhedra in Bi_4_O_5_I_2_ were performed. The details of the calculations are also provided in ESI.† The results and details of the calculations are provided in Table ESI-2.† It proves that the direction of the dipole moment of Bi_4_O_5_I_2_ powder is along [0 −1 0]. Due to the increased dipole moments, Bi_4_O_5_I_2_ has better separation efficiency of electron–hole pairs. The polarization promotes the separation of photogenerated electron–hole pairs and mitigates the charge recombination, thus resulting in high photocatalytic activity. The calculated results are in accordance with those obtained *via* the photocatalytic degradation experiments.

### 3.8. PC and EIS analyses

**Fig. 5:**
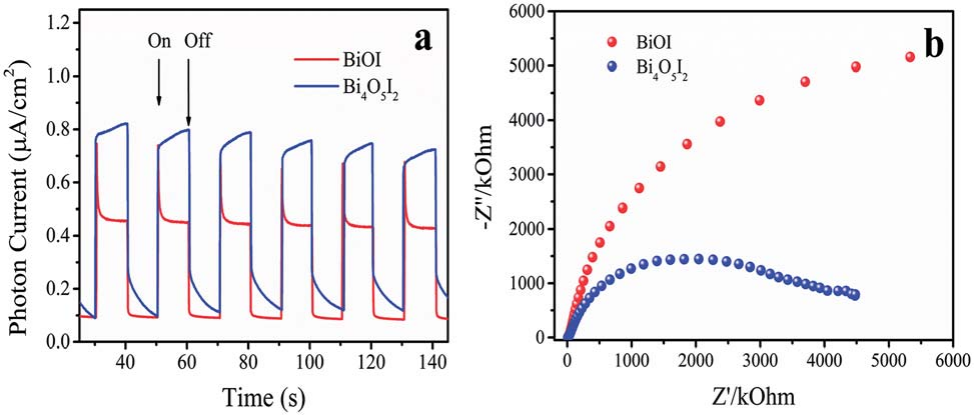
(a) Transient photocurrent spectra and (b) electrochemical impedance spectra of BiOI and Bi_4_O_5_I_2_.

The separation of photoinduced electron–hole pairs and the charge transfer processes in Bi_4_O_5_I_2_ were investigated by transient photocurrent (TPC) response and electrochemical impedance spectra (EIS), respectively. Fig. 5a shows the instantaneous photocurrent response of BiOI and Bi_4_O_5_I_2_ catalysts. As can be seen from Fig. 5a, due to the action of dipole moments, the photocurrent of Bi_4_O_5_I_2_ is significantly higher than that of BiOI, which promotes the effective separation of photogenerated carriers, thereby resulting in an increase in photocurrent. To better understand the PC measurement results, the EIS spectrum in the form of a Nyquist plot are shown in Fig. 5b. It reflects the recombination rate of electrons and holes during the photoreaction. The smaller arc radius reflects the lower resistance of the interface charge transfer.^49^ It is clearly shown that Bi_4_O_5_I_2_ has a smaller arc radius, lower chemical impedance and faster charge transport rate. This is consistent with the abovementioned characterization results.

### 3.9. PL analysis

Photoluminescence (PL) technique was employed herein to further confirm the transfer and recombination of photogenerated electrons and holes for BiOI and Bi_4_O_5_I_2_ during the process of photocatalysis. Fig. S3c† shows that Bi_4_O_5_I_2_ sample exhibits much lower intensity than BiOI sample, which suggests that Bi_4_O_5_I_2_ sample possesses lower recombination rate of the photogenerated charge carriers. It is consistent with the results of PC and EIS analyses.

### 3.10. Mott–Schottky plot analysis

Mott–Schottky plots (*vs.* SCE) for Bi_4_O_5_I_2_ are shown in Fig. S3d.† It is known that the conduction bands of the n-type semiconductors are 0–0.1 eV higher than that of the flat potentials, which could be estimated with Mott–Schottky plots. The flat potential is calculated to be −0.75 V *versus* the saturated calomel electrode (SCE), which is equivalent to −0.51 V *versus* the normal hydrogen electrode (NHE). Therefore, the *E*_CB_ of Bi_4_O_5_I_2_ was estimated to be −0.61 eV *vs.* NHE. *E*_VB_*vs.* NHE can further be calculated from the equation *E*_VB_ = *E*_g_ + *E*_CB_, which is estimated to be 1.78 eV *vs.* NHE.

### 3.11. Mechanism analysis

**Fig. 6:**
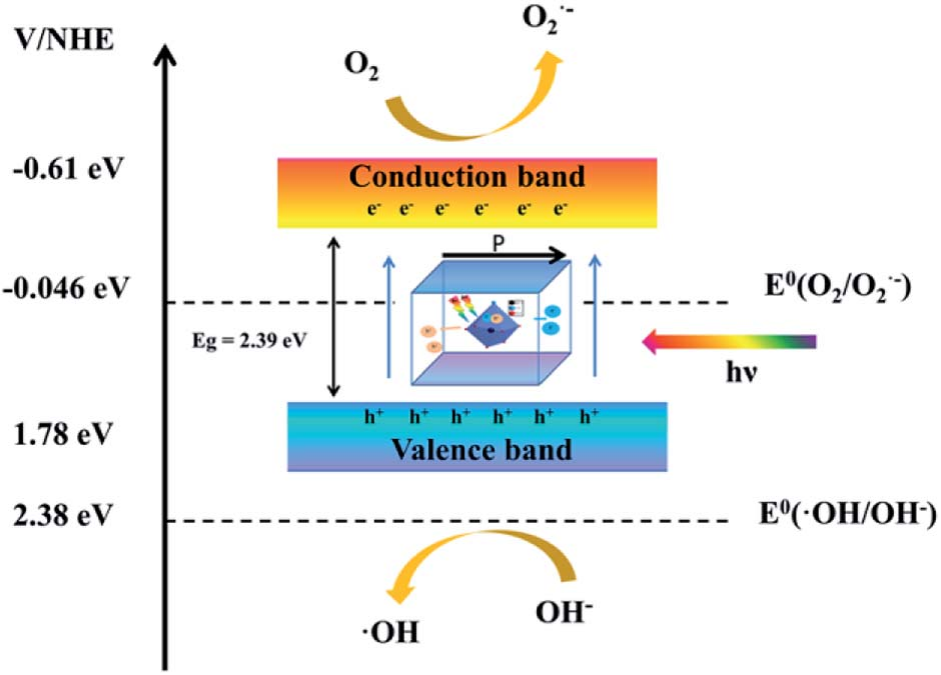
Schematic of the energy band of Bi_4_O_5_I_2_ as well as charge migration and separation caused by visible light irradiation.

Fig. 6 shows a schematic diagram of the band structure and charge migration and separation caused by visible light irradiation. Due to the action of dipole moments, Bi_4_O_5_I_2_ noncentrosymmetric polarized structure promotes the separation of photogenerated electron–hole pairs, which leads to efficient photocatalytic activity. On the other hand, based on the trapping and Mott–Schottky experiment, h^+^ and ·O^2−^ play the role of major active radicals during photodegradation. The possible reason is that a part of ·O^2−^ is converted into ·OH. In addition, *E*_CB_ of Bi_4_O_5_I_2_ is estimated to be −0.61 eV *vs.* NHE, which is lower than that of *E*_0_ (O_2_/O_2_˙^−^ = −0.046 eV *vs.* NHE). Therefore, the photogenerated electrons on the CB of Bi_4_O_5_I_2_ could be trapped by molecular oxygen to generate ·O^2−^ under visible light.

However, its valence band is more negative than *E*_0_ (OH^−^/·OH = 2.38 eV *vs.* NHE). The result is corresponding to the trapping experiments. The mechanism of the active radical production is proposed as follows:e^−^ + O_2_ = ·O_2_^−^2e^−^ + O_2_ + 2H^+^ = H_2_O_2_·O_2_^−^ + H_2_O_2_ →·OH + OH^−^ + O_2_h^+^ + H_2_O = ·OH + H^+^

### 3.12. PDOS and band-resolved analysis

**Fig. 7:**
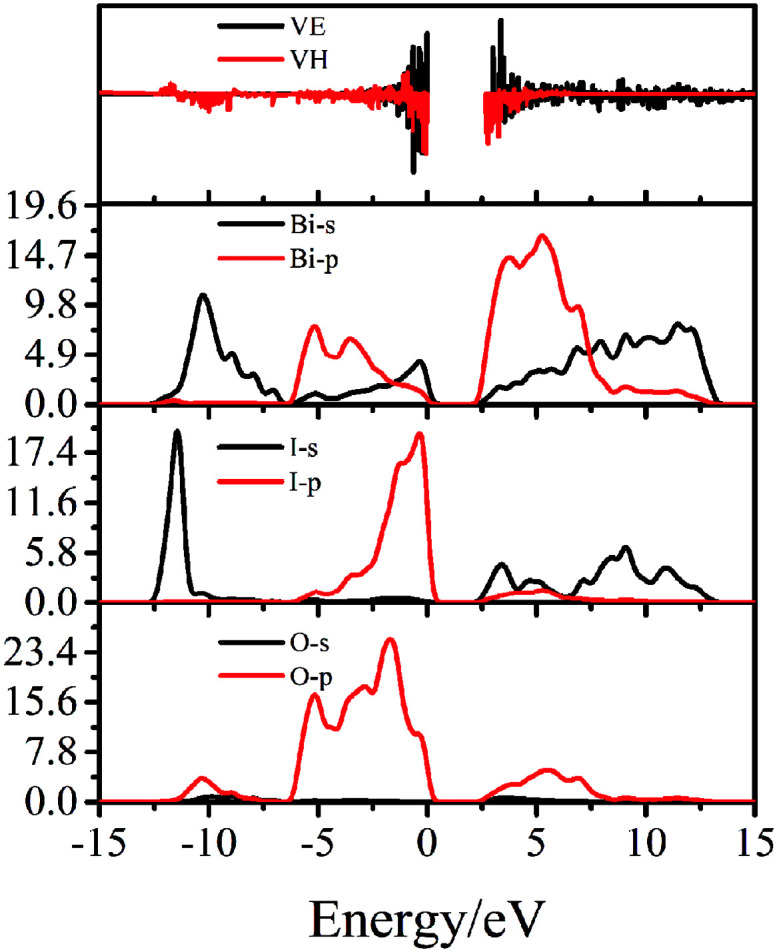
The PDOS and band-resolved VE and VH processes.

To clearly understand the essence of photocatalysis and NLO properties, the partial density of states (PDOSs) of Bi_4_O_5_I_2_ were calculated. For Bi_4_O_5_I_2_, both the O 2p and I 5p states contributed to the top of the VB and Bi 6p and O 5p states dominated most of the bottom of the CB. The SHG efficiencies are strongly influenced by the direction and the magnitude of the dipole moments of polar structure units.^50^ The calculated SHG coefficients of Bi_4_O_5_I_2_ are *d*_22_ = −7.82 pm V^−1^ and ∼20 KDP (*d*_22_) (where KDP, KH_2_PO_4_, *d*_36_ ≈ 0.39 pm V^−1^, which means it should have large SHG responses). Herein, we considered the SHG efficiencies of Bi_4_O_5_I_2_. However, because of the very small size of the Bi_4_O_5_I_2_ powder particles, the SHG efficiencies could not be directly accommodated in the experiment. We could not test the results of theoretical predictions experimentally. Therefore, we planned to grow bigger Bi_4_O_5_I_2_ crystals and further study and explore the NLO properties of Bi_4_O_5_I_2_. In addition, we used the band-resolved method to further explain the contributions of orbitals to the SHG response. The virtual-electron (VE) and virtual-hole (VH) contributions to the total SHG coefficients were obtained. Based on the band-resolved results shown in Fig. 7, these VE and VH contributions are 61.6% and 38.4%, respectively. It indicates that the SHG effect may be due to the VE and VH processes.

## 4. Conclusions

In summary, exploration of the versatility of materials to increase the utilization of materials is a hot research topic nowadays. Herein, Bi_4_O_5_I_2_ powders have been fabricated *via* a facile solvothermal method. Under the light irradiation for 60 min, 94% of MO and MB were degraded over Bi_4_O_5_I_2_ samples. It is noteworthy that for the degradation of methyl orange, the photocatalytic activity of Bi_4_O_5_I_2_ is about 10 times that of BiOI. Due to the action of dipole moments, charge separation of the photogenerated electron–hole pairs occurs and thus enhances the photocatalytic activity. In addition, increased dipole moment due to the stereochemical activity of lone pairs induces a high second harmonic generation response. Moreover, theoretical calculation and experiment results reveal that Bi_4_O_5_I_2_ would have potential applications in photocatalytic and NLO fields.

## Conflicts of interest

There are no conflicts of interest to declare.

## Supplementary Material

RA-009-C8RA08984A-s001
