# Supplementary material for: Multifunctional property exploration: Bi4O5I2 with high visible light photocatalytic performance and a large nonlinear optical effect
Source: RSC Adv. 2019 Feb 5;9(8):4539–44. doi: 10.1039/c8ra08984a (PMC9060602; doi:10.1039/c8ra08984a)
Supplement: RA-009-C8RA08984A-s001 [file RA-009-C8RA08984A-s001.pdf]

Electronic Supplementary Material (ESI) for RSC Advances.
This journal is © The Royal Society of Chemistry 2019

Research Highlights of "**Multifunctional property exploration: $Bi_4O_5I_2$ with highly visible light photocatalytic performance and large nonlinear optical effect**"

Fig. S1 EDS patterns of BiOI (a) and $Bi_4O_5I_2$ (b) samples

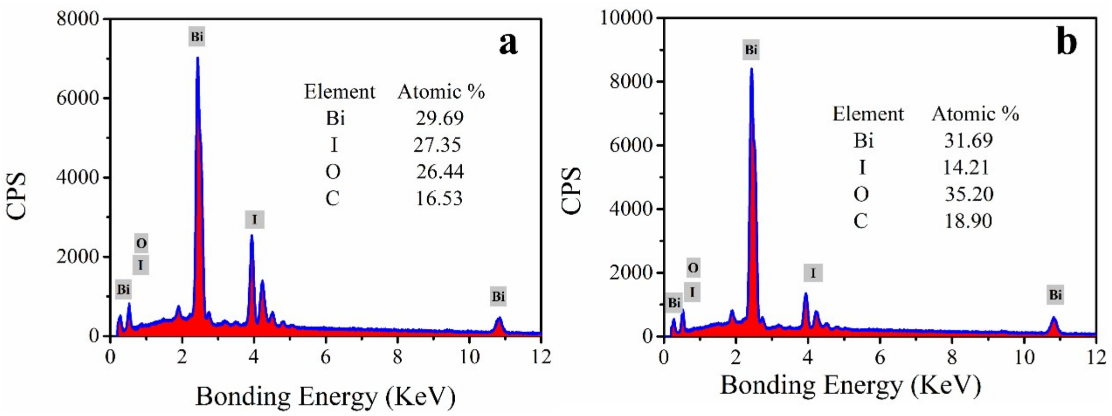

Fig. S2 Photocatalytic activity of Blank (the initial concentration of 10 mg/L, catalyst is 50mg and without light), $Bi_4O_5I_2$, and P25 for degradation of MO under visible light irradiation (the initial concentration of 10 mg/L and amount of catalyst is 50mg) (a), effect of MO initial concentration on the photocatalytic performances of $Bi_4O_5I_2$ (b), photocatalytic degradation of MO, RhB, MB over BiOI and $Bi_4O_5I_2$ samples, respectively (c), photocatalytic activity of $Bi_4O_5I_2$ for degradation of norfloxacin under visible light irradiation (d)

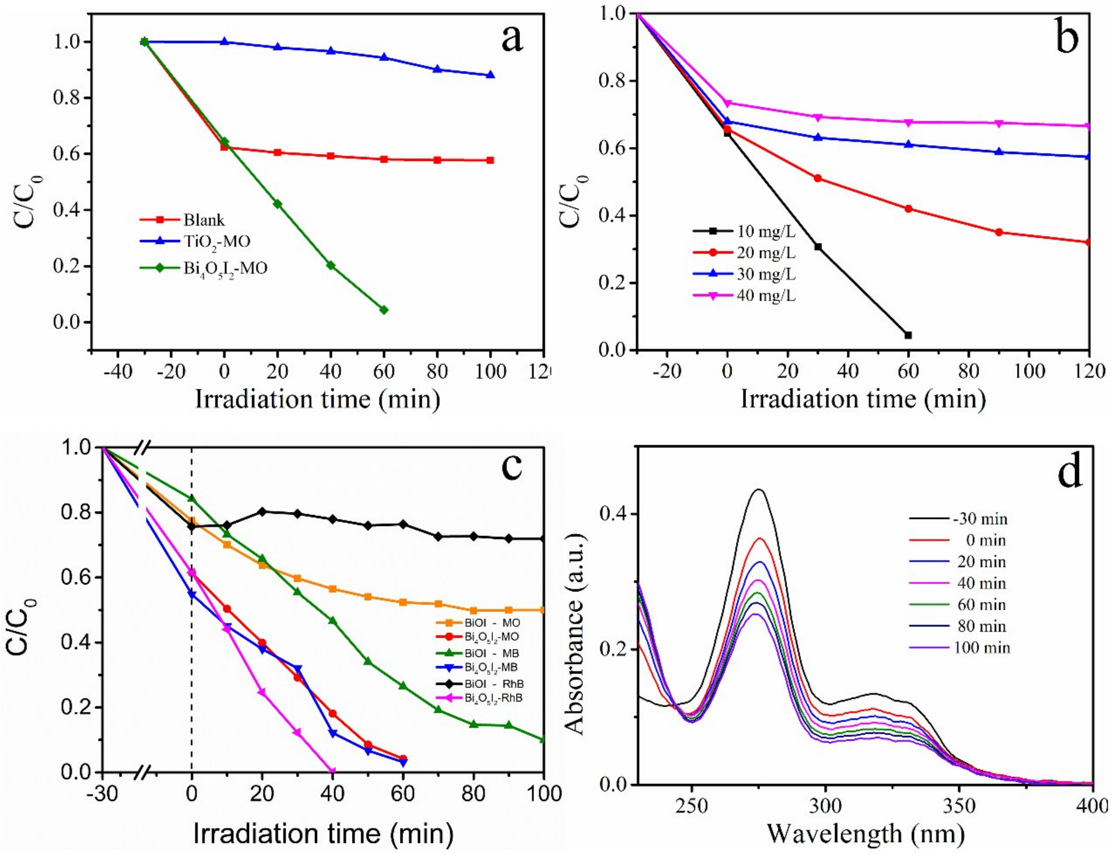

Fig. S3 Free radicals trapping experiment of $Bi_4O_5I_2$ photocatalysts under visible light irradiation (a), Recycling property of $Bi_4O_5I_2$ photocatalyst (b), PL spectra of BiOI and $Bi_4O_5I_2$ (c), Mott-Schottky plots of the $Bi_4O_5I_2$ samples (d)

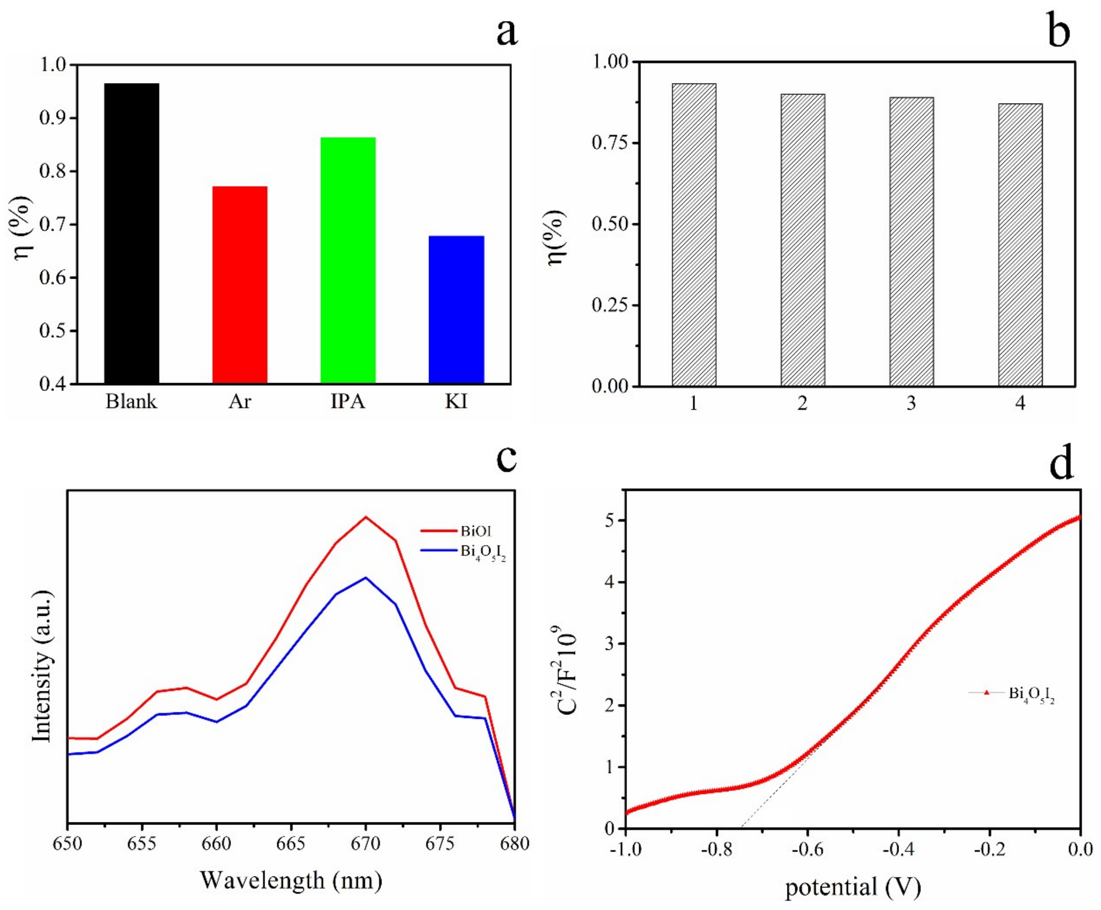

Table ESI-1. Pseudo-first-order kinetics parameter of MO

	Pseudo-First-Order reaction		
catalyst	k_{app} (min^{-1})	R^2	Stansard error
BiOI	0.0042	0.8636	0.0005
$Bi_4O_5I_2$	0.0443	0.9237	0.0052

An internal polar electric field is constructed along the *ab* plane in the crystal structure of $Bi_4O_5I_2$ and the alignment of BiO_4I_4, BiO_5I_3, BiO_6I, BiO_3I_4 and BiO_3I_5 polyhedron in an individual polyhedra.

The bond-valence-sum Vi for each atom i is defined as

$$V_i = \sum_j S_{ij} = \sum \exp[\frac{R_0 - R_{ij}}{B}]$$

where B is a constant with value 0.37. R0 and Rij represent the reference and actual lengths of the bond i-j that the atom i makes with the surrounding atoms j, respectively, and Sij is the corresponding bond valence. R represents the difference between the "centroids" of the positive (r^+) and negative charges r^-).

$$R = r^+ - r^-$$

For a given Bii-Oj bond with the actual bond length Rij, the nuclear charges of Bi and O are 83 and 8, respectively

$$(83 - V_i) \times r^- = (8 + S_{ij}) \times (R_{ij} - r^-)$$

Then, in units of Debye, in which R is measured by Å and the charge by statcoulomb, the net bond dipole moment μ_{ij} of the Bii-Oj bond is then calculated using the expression

$$\mu_{ij} = n_{ij} e R$$
$$n_{ij} = (83 - V_i) + (8 + S_{ij})$$

where n_{ij} is the number of electrons forming the Bii-Oj, and e the electron charge (i.e., 4.8× 10^{-10}statcoulombs in cgs unit)

Table ESI-2. Dipole moments of BiO_4I_4, BiO_5I_3, BiO_6I, Bi(1)O_3I_4 , BiO_3I_5 polyhedra in $Bi_4O_5I_2$ crystal

Polyhedral Unit	Bi(1)O_4I_4	Bi(3)O_4I_4	Bi(1)O_5I_3	Bi(3)O_5I_3	Bi(1)O_6I	Bi(3)O_6I	Bi(1)O_3I_4	Bi(1)O_3I_5	total
Polyhedral Moment (esu·cm/Å^3)	0.149	0.141	0.117	0.118	0.069	0.065	0.163	0.12	
X	-18.75	-17.4	-22.08	-13.37	9.79	-5.09	-14.29	-1.74	
Y	0.76	0.82	3.3	-7.37	-6.68	5.51	2.28	-3.32	
Z	26.71	25.64	12.79	-8.45	11.42	25.99	-33.42	27.62	
Polyhedral Unit	Bi(2)O_4I_4	Bi(4)O_4I_4	Bi(2)O_5I_3	Bi(4)O_5I_3	Bi(2)O_6I	Bi(4)O_6I	Bi(2)O_3I_4	Bi(2)O_3I_5	
Polyhedral Moment (esu·cm/Å^3)	0.149	0.141	0.117	0.118	0.069	0.065	0.163	0.12	
X	18.75	17.4	22.08	13.37	-9.79	5.09	14.29	1.74	0
Y	0.76	0.82	3.3	-7.37	-6.68	5.51	2.28	-3.32	-9.4
Z	-26.71	-25.64	-12.79	8.45	-11.42	-25.99	-33.42	-27.62	0
